# A comparative study based on deformable image registration of the target volumes for external-beam partial breast irradiation defined using preoperative prone magnetic resonance imaging and postoperative prone computed tomography imaging

**DOI:** 10.1186/s13014-019-1244-x

**Published:** 2019-03-05

**Authors:** Ting Yu, Jian Bin Li, Wei Wang, Min Xu, Ying Jie Zhang, Qian Shao, Xi Jun Liu, Liang Xu

**Affiliations:** 1 School of Medicine and Life Sciences, University of Jinan-Shandong Academy of Medical Sciences, Jinan, Shandong province China; 2 Department of Radiation Oncology, Shandong Cancer Hospital Affiliated to Shandong University, Shandong Academy of Medical Sciences, 440 Jiyan Road, Jinan, 250117 China; 3 Department of Medical Imaging, Shandong Cancer Hospital Affiliated to Shandong University, Shandong Academy of Medical Sciences, Jinan, Shandong province China

**Keywords:** Preoperative diagnostic magnetic resonance image, Postoperative prone computed tomography simulation image, Deformable image registration, Target volume comparison, External-beam partial breast irradiation

## Abstract

**Background:**

To explore the differences and correlations between the target volumes defined using preoperative prone diagnostic magnetic resonance imaging (MRI) and postoperative prone computed tomography (CT) simulation imaging based on deformable image registration (DIR) for external-beam partial breast irradiation (EB-PBI) after breast-conserving surgery (BCS).

**Methods:**

Eighteen breast cancer patients suitable for EB-PBI were enrolled. Preoperative prone diagnostic MRI and postoperative prone CT scan sets for all the patients were acquired during free breathing. Target volumes and ipsilateral breast were all contoured by the same radiation oncologist. The gross tumor volume (GTV) delineated on the preoperative MRI images was denoted as the GTV_preMR_ and the tumor bed (TB) delineated on the postoperative prone CT images was denoted as the GTV_postCT_. The MIM software system was used to deformably register the MRI and CT images.

**Results:**

When based on the coincidence of the compared target centers, there were statistically significant increases in the conformity index (CI) and degree of inclusion (DI) values for GTV_postCT_-GTV_preMR_, GTV_postCT_-CTV_preMR + 10_, CTV_postCT + 10_-GTV_preMR,_ and CTV_postCT + 10_-CTV_preMR + 10_ when compared with those based on the DIR of the thorax (*Z* = − 3.724, − 3.724, − 2.591, − 3.593, all *P <* 0.05; *Z* = -3.724, − 3.724, − 3.201, − 3.724, all *P <* 0.05, respectively).

**Conclusions:**

Although based on DIR, there was relatively poor spatial overlap between the preoperative prone diagnostic MRI images and the postoperative prone CT simulation images for either the whole breast or the target volumes. Therefore, it is unreasonable to use preoperative prone diagnostic MRI images to guide postoperative target delineation for EB-PBI.

## Background

Breast-conserving therapy (BCT), which involves a wide local excision followed by radiotherapy to the whole breast, has become the standard treatment for early-stage breast cancer [1]. However, for patients with a low risk of recurrence, accelerated partial breast irradiation (APBI) is now gaining acceptance as an alternative to whole breast irradiation (WBI) for early-stage cancer [2–6]. In addition, external-beam partial breast irradiation (EB-PBI) is an important approach to APBI. Polgar et al. [2] have reported that the efficacy of EB-PBI is equivalent to that of WBI. However, there are conflicting data regarding the acute and late toxicity of APBI. An Italian randomized trial has indicated [7] that the rates of Grades 1 and 2 acute skin toxicity in a APBI cohort were remarkably lower than those in a WBI group with decreases of 17 and 18.2%, respectively. However, in a prospective trial of 2135 patients from Canada [8], poor cosmesis at 3 years was significantly increased among those treated with APBI compared with WBI treatment, with 29% vs 17% as determined by trained nurses and 26% vs 18% as determined by the patients. Meanwhile, the rates of Grades 1 and 2 toxicity in the EB-PBI patients were also significantly higher than those in the WBI group.

Hence, based on these results, researchers are rethinking all aspects of postoperative EB-PBI. A potential factor that explains the increase in toxicity observed in the APBI group is the irradiation of a larger volume of breast tissue in those patients with poor cosmesis. Whether to ensure therapeutic efficacy or to reduce toxicity and side effects, an essential prerequisite for APBI is the accurate delineation of the target volume. However, defining the target in postoperative EB-PBI varies widely depending on the specimen volume, seroma size, clarity, surgical clips, simulation image, inter-observer variability [9] and other aspects. In addition, there is a volumetric difference for EB-PBI between the prone and supine positions [10]. Moreover, preoperative EB-PBI might be an effective approach to reducing the target volume compared to that in postoperative EB-PBI. It has been reported that both the gross tumor volume (GTV) and planning target volume (PTV) are significantly smaller in preoperative EB-PBI than in postoperative EB-PBI [11, 12].

Preoperative image-guided techniques have been considered effective tools for improving the detection of tumors [13], and due to its high spatial resolution, preoperative magnetic resonance imaging (MRI) has the advantages of detecting occult tumors and providing additional valuable information regarding the primary tumor [14, 15]. At present, there are few reports that focus on the feasibility of preoperative prone diagnostic MRI in guiding postoperative target delineation for prone EB-PBI. Therefore, the aim of this study was to provide a reference for how to use preoperative diagnostic MRI to guide the delineation of postoperative EB-PBI in the prone position.

## Methods

### Patient selection

Breast cancer patients who were suitable for EB-PBI after BCS were enrolled in this study. All the patients underwent preoperative diagnostic MRI in the prone position. Patients who had oncoplastic BCS were excluded from the trial, and equal or more than 5 surgical clips (2 mm in diameter) were used to mark the boundaries of the lumpectomy cavity. All of the enrolled patients had either no seroma or a seroma clarity score of ≦3 in the surgical cavity. None of the patients had chronic lung disease, and all exhibited normal arm movement after surgery. This research was performed in accordance with the relevant regulations, and all the patients in our research joined this study with informed consent and voluntarily underwent prone 3D CT simulation scanning. The study was approved by the institutional research ethics board of the Shandong Tumor Hospital Ethics Committee.

### Image simulation and acquisition

Patients underwent preoperative prone diagnostic MRI that was performed with a Philips Achieva 3.0-T scanner (Amsterdam, Netherlands). The diagnostic MRI protocol began with preliminary imaging using fast-spin echo sagittal T2 with fat saturation, T2 weighted (T2w) turbo spin echo (TSE) with fat suppression [spectral adiabatic inversion recovery (SPAIR)]and axial T1 sequences. This was followed by dynamic high resolution simultaneous imaging of both breasts using the THRIVE sequence with 8 dynamic scans with fat saturation, performed after intravenous administration of a contrast agent (gadopentetate dimeglumine, 0.1 mmol/kg). Postprocessing consisted of 2 series of subtraction images. The total acquisition time of the preoperative diagnostic MRI protocols was 18 min. All the patients were placed in the prone position on the dedicated bilateral breast coil with no degree of incline. The coil contained two apertures open all sides to allow the bilateral breasts to hang freely away from the chest wall. The hands were naturally extended and placed on both sides of the head.

While undergoing postoperative CT simulation scanning with standard resolution, matrix 512 × 512, the patients were placed in the prone position on a dedicated treatment board (CIVCO Horizon™ Prone Breast Bracket- MTHPBB01) with no degree of incline using an arm support (with both arms above the head). The board contained an open aperture on one side to allow the ipsilateral breast to hang freely away from the chest wall. The MRI and CT images that were transferred to the MIM version 6.7.6 software (Cleveland, USA) were 3-mm thick.

### Target volume delineation

**Fig. 1:**
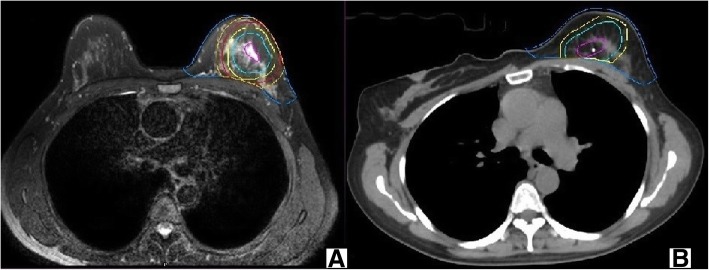
The picture of target volumes based on preoperative prone diagnostic MRI or postoperative prone simulation CT (**a** preoperative diagnostic MRI; **b** postoperative simulation CT)

All structures were delineated by the same radiation oncologist on both the preoperative diagnostic MRI and postoperative CT simulation images using the MIM system. MRI delineations were performed based on the preoperative T2WI images with voxel size 1 mm × 1.25 mm × 3 mm; the gross tumor was delineated based on hyperintense T2WI area (excluding the hypersignal gland around the primary tumor) and denoted as the GTV_preMR,_. The clinical target volumes (CTVs) consisted of the GTV_preMR,_ plus 10-mm and 20-mm margins and were denoted as the CTV_preMR + 10_ and CTV_preMR + 20_, respectively. All of the CTVs were limited to 5 mm from the skin surface and the gland-pectoralis interface. The PTVs were expanded by 15-mm and 25-mm margins from the GTV_preMR,_ and were denoted as the PTV_preMR + 15_ and PTV_preMR + 25_, respectively. All of the PTVs were limited to 5 mm from the skin surface and lung-chest wall interface. While delineating the CTV_Breast-MRI_, the delineator considered referenced breast at time of MRI, including the apparent MRI glandular breast tissue and incorporating consensus definitions of anatomical range. After delineating, the MRI physician was asked to confirmed the target volumes defined using the preoperative diagnostic MRI (Fig. 1a).

On the postoperative prone simulation CT images, the tumor bed was delineated based on the surgical clips alone and was defined as the GTV_postCT_. The CTV_postCT + 10_ was created by adding 10 mm to the GTV_postCT_ and was limited to 5 mm from the skin surface and the gland-pectoralis interface. The PTV_postCT + 15_ was produced by equally extending the GTV_postCT_ by a 15-mm margin and was limited to 5 mm from the skin surface and the lung-chest wall interface. The ipsilateral breast was contoured over the obtained MRI and CT images. And the CTV_Breast-CT_ was delineated by considering referenced breast at time of CT, including the apparent CT glandular breast tissue and incorporating consensus definitions of anatomical range (Fig. 1b).

### Deformable image registration

**Fig. 2:**
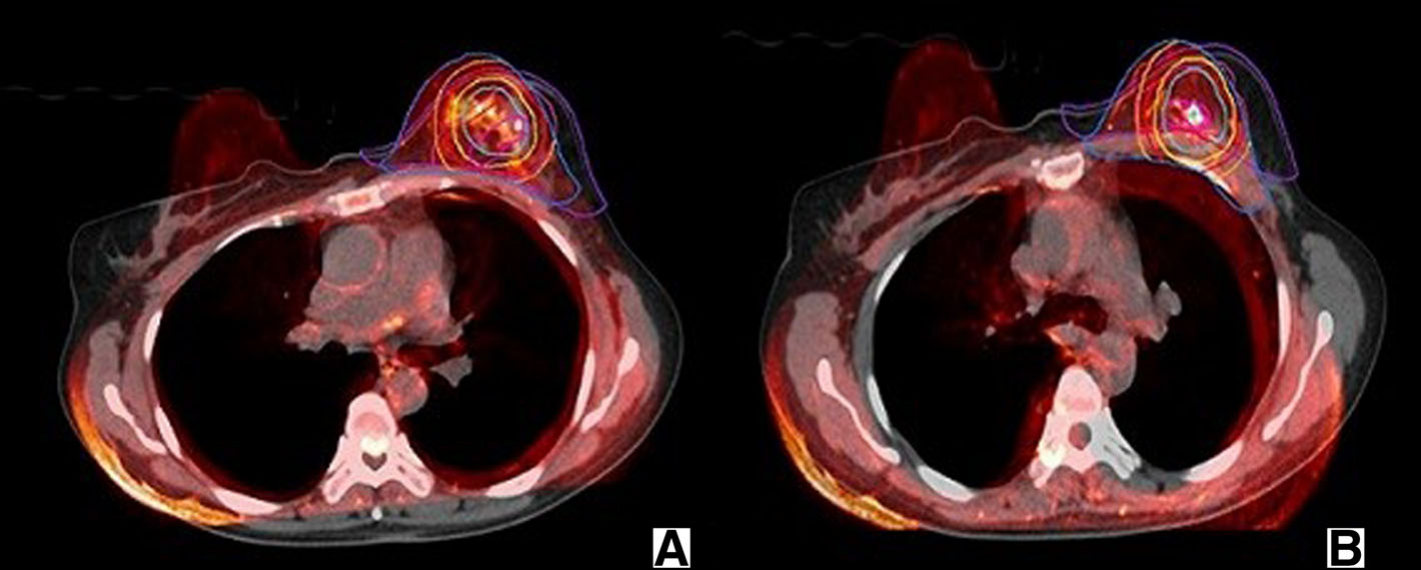
The picture of DIR based on the thorax and DIR based on the center-coincidence of the GTV_preMR_ - GTV_postCT_ (**a** based on the thorax; **b** based on the center-coincidence of the GTV_preMR_ -GTV_postCT_)

This study applied the MIM system to perform the deformation registration. The VoxAlign Deformation Engine™ provided a registration algorithm for converting local registration into deformation registration in different modality images registrations. Meanwhile, the MIM system was a commercial software, so the algorithm details were not public. First, the main sequence and a subordinated sequence were selected for rigid registration. On this basis, the automatic deformation registration was implemented with set reference points, including the thorax and the center-coincidence of the compared targets. During the registration process of this study, the prone CT simulation was set to the main sequence, and the MRI T2WI image was used as the subordinated sequence. After the automatic deformation registration was completed, the Reg Reveal and Reg Refine tools were used to evaluate and revise the registration quality of the images to achieve the best visual effect (Fig. 2). The Reg Reveal tool was used for evaluating an image’s final deformable registration results in the primary area of concern and the Reg Refine tool would only be used in the event that, while evaluating the initial deformation with Reg Reveal, it was determined a poor alignment was identified that needs to fixed.

### Parameter evaluation

The target volumes defined using the preoperative diagnostic MRI and postoperative prone CT images were calculated separately. In addition, the correlations between the target volumes defined using the preoperative diagnostic MRI and the corresponding target volumes based on postoperative prone CT images were evaluated respectively.

The degree of inclusion (DI), the conformity index (CI) and Dice’s similarity coefficient (DSC) were calculated for the CTV_Breast-MRI_ and CTV_Breast-CT_, the GTV_postCT_ and GTV_preMR,_ the GTV_postCT_ and CTV_preMR + 10_, the CTV_postCT + 10_ and GTV_preMR,_ and the CTV_postCT + 10_ and CTV_preMR + 10_. The DI was defined as follows:

$$ \mathrm{DI}\ \left(\mathrm{A},\mathrm{B}\right)=\frac{A\cap B}{A} $$

The definition of the DI of volume A included in volume B [DI (A in B)] was the percentage of the overlap between volume A and B in volume A [16]. The CI of volume A and B [CI (A, B)] was computed according to Struikmans et al. [17] The formula was as follows:

$$ \mathrm{CI}\ \left(\mathrm{A},\mathrm{B}\right)=\frac{A\cap B}{A\cup B} $$

which is defined as the ratio of the intersection of A with B to the union of A and B. DSC [18] is a commonly used metric in medical imaging and contouring studies and is defined as follows:

$$ \mathrm{DSC}=\frac{2\left(A\cap B\right)}{A+B}. $$

The three-dimensional coordinates of the targets were recorded for each patient. Next, the displacements between the targets in the left-right (LR), anterior-posterior (AP) and superior-inferior (SI) directions were obtained and were defined as Δx, Δy and Δz, respectively. The distance between of the centers of mass (COMs) of the targets was calculated using the following formula:

$$ \varDelta \mathrm{V}=\Big(\varDelta {\mathrm{x}}^2+\varDelta {\mathrm{y}}^2+\varDelta {z}^{2\Big)1/2} $$

### Statistical analyses

Statistical analysis was performed using the SPSS 19.0 software (IBM Corporation, Armonk, NY, USA). The data that did not follow a normal distribution are described using medians and ranges. The Wilcoxon signed-rank test was used to compare the target volumes and relevant parameters. The Spearman rank correlation analysis was performed to establish the relevance of differences between the target volumes. The data were considered statistically significant at *P* < 0.05.

## Results

### Patient characteristics

The study population consisted of 30 patients with early-stage breast cancer who were suitable for EB-PBI after BCS from July 2016 to April 2017. Eighteen of the 30 patients who underwent preoperative diagnostic MRI enrolled in this study. The patients had a median age of 43 years (range, 39–69 years) and had cancer of the breast with a pathological stage of T1-T2N0M0. Seven of the 18 patients had left-sided breast cancer, and the remaining eleven had right-sided breast cancer. The patients underwent a lumpectomy, which was performed with a circumferential margin of at least 1.0 cm [19], with sentinel lymph node dissection (SLND) or axillary lymph node dissection (ALND), and tumor-negative margins were ensured during a single operation. The patient and tumor characteristics are presented in Table 1.

**Table 1:** Patient and tumor characteristics

Variables	Values
Age, years	
Median	43
Range	39–69
Tumor size	
≥ 10 mm < 20 mm	10
≥ 20 mm < 30 mm	8
Breast side	
Left	7
Right	11
Localization of tumor bed	
UOQ	11
LOQ	1
Central portion of breast	0
UIQ	2
LIQ	4
Tumor characteristics	
Ductal carcinoma in situ	1
Invasive ductal carcinoma	15
Invasive lobular carcinoma	1
Cribriform carcinoma	2

Abbreviations: *UOQ* upper outer quadrant, *LOQ* lower outer quadrant, *UIQ* upper inner quadrant, *LIQ* lower inner quadrant

### Comparison of the target volumes and correlation analysis

The target volumes defined using preoperative prone MRI and postoperative prone CT are listed in Table 2. The median GTV_preMR,_ was 12.58 cm^3^ less than the median GTV_postCT_ (*Z* = -3.593, *P* = 0.000). After expanding the GTV with the described margins, the median values of the CTV_postCT + 10_ and PTV_postCT + 15_ were both significantly larger than those of the CTV_preMR + 10_ and PTV_preMR + 15_, respectively (*Z* = -3.593, − 2.983, both *P <* 0.05). Moreover, the median volume variability between the CTV_postCT + 10_ and CTV_preMR + 20_ and between the PTV_postCT + 15_ and PTV_preMR + 25_ was statistically significant (*Z* = -2.722, − 2.853, both *P* < 0.05). A statistically significant positive correlation was found between the GTV_preMR,_ and GTV_postCT_, the CTV_preMR + 10_ and GTV_postCT_, and the CTV_preMR + 10_ and CTV_postCT + 10_ (*r* = 0.518, 0.474, 0.498; *P* = 0.028, 0.042, 0.047, respectively). However, there was no significant correlation between the PTV_preMR + 15_ and PTV_postCT + 15_ or the PTV_preMR + 25_ and PTV_postCT + 15_ (Table 3).

**Table 2:** Target volume defined using preoperative prone MRI and postoperative prone CT (cm^3^)

Target volume	Median	Range
GTV_preMR_	4.64	1.34~19.06
CTV_preMR + 10_	34.33	18.56~87.14
PTV_preMR + 15_	71.05	41.48~150.57
CTV_preMR + 20_	105.27	62.08~205.17
PTV_preMR + 25_	162.00	92.27~260.95
GTV_postCT_	17.22	9.04~42.46
CTV_postCT + 10_	53.46	22.69~118.21
PTV_postCT + 15_	118.01	79.29~227.08

Abbreviations: *GTV* gross tumor volume, *CTV* clinical target volume, *PTV* planning target volume, *TB* tumor bed

**Table 3:** Correlation between the target volumes defined using preoperative prone MRI and postoperative prone CT

Target volume	*r*-value	*P*-value
GTV_postCT-_ GTV_preMR_	0.518	0.028
GTV_postCT-_ CTV_preMR + 10_	0.483	0.042
GTV_postCT -_CTV_preMR + 20_	0.399	0.101
CTV_postCT + 10-_CTV_preMR + 10_	0.474	0.047
CTV_postCT + 10-_CTV _preMR + 20_	0.498	0.053
PTV_postCT + 15_- PTV_preMR + 15_	0.401	0.099
PTV _postCT + 15_-PTV _preMR + 25_	0.377	0.123

Abbreviations: *GTV* gross tumor volume, *CTV* clinical target volume, *PTV* planning target volume, *TB* tumor bed

### Comparison of the parameters of the target volumes defined using MRI and CT

When based on the DIR of the thorax, the median values of the CI, DI and DSC between the CTV_Breast-MRI_ and CTV_Breast-CT_ were 0.56, 0.82 and 0.71, respectively. The distance between the COM of the CTV_Breast-MRI_ and CTV_Breast-CT_ was 1.81 cm. When based on the DIR of the thorax, the median CIs for GTV_postCT_-GTV_preMR,_ GTV_postCT_- CTV_preMR + 10_, CTV_postCT + 10_-GTV_preMR,_ and CTV_postCT + 10_-CTV_preMR + 10_ were slightly lower than those based on the center-coincidence of the GTV_preMR_ and GTV_postCT_; the median CI values for these volumes were 0.02, 0.07, 0.04 and 0.17; 0.19, 0.31, 0.05 and 0.38, respectively (*Z* = − 3.724, − 3.724, − 2.591, − 3.593, respectively; all *P <* 0.05). The DI and DSC median values for the GTV_postCT_-GTV_preMR_, the GTV_postCT_-CTV_preMR + 10_, the CTV_postCT + 10_-GTV_preMR_ and the CTV_postCT + 10_-CTV_preMR + 10_ were generally low; however, there were statistically significant increases in these parameters based on the center-coincidence of the GTV_preMR_ and GTV_postCT_ when compared with those based on the DIR of the thorax (*Z* = − 3.724, − 3.724, − 3.201, − 3.724, all *P <* 0.05; *Z* = -3.724, − 3.724, − 2.591, − 3.636, all *P <* 0.05, respectively, Table 4).

**Table 4:** Parameter evaluation of the target volume defined using preoperative prone MRI and postoperative prone CT based on the DIR of the thorax or target center

Parameters	On DIR of the thorax	On DIR of the target center	*Z*-value	*P*-value
GTV_postCT -_GTV_preMR_				
CI	0.02 (0.00~0.18)	0.19 (0.06~0.48)	−3.724	0.000
DI	0.08 (0.00~0.54)	0.85 (0.48~1.00)	−3.724	0.000
DSC	0.03 (0.00~0.31)	0.32 (0.11~0.65)	−3.724	0.000
ΔV	2.71 (0.55~7.21)	0.06 (0.02~1.91)	-3.724	0.000
GTV_postCT -_ CTV_preMR + 10_				
CI	0.07 (0.00~0.23)	0.31 (0.17~0.48)	-3.724	0.000
DI	0.27 (0.00~0.85)	0.86 (0.38~1.00)	-3.724	0.000
DSC	0.13 (0.00~0.38)	0.47 (0.30~0.65)	-3.724	0.000
ΔV	2.67 (1.00~6.42)	0.14 (0.04~1.96)	-3.724	0.000
CTV_postCT + 10_- GTV_preMR_				
CI	0.04 (0.00~0.16)	0.05 (0.01~0.20)	−2.591	0.010
DI	0.66 (0.00~1.00)	1.00 (0.84~1.00)	−3.201	0.001
DSC	0.07 (0.00~0.27)	0.09 (0.03~0.34)	−2.591	0.010
ΔV	2.54 (0.55~6.57)	0.28 (0.04~1.70)	−3.724	0.000
CTV_postCT + 10_- CTV_preMR + 10_				
CI	0.17 (0.00~0.46)	0.38 (0.13~0.67)	−3.593	0.000
DI	0.43 (0.00~0.90)	0.89 (0.65~0.99)	−3.724	0.000
DSC	0.29 (0.00~0.63)	0.55 (0.24~0.80)	−3.636	0.000
ΔV	2.67 (0.00~6.44)	0.21 (0.05~1.75)	−3.724	0.000

Abbreviations: *CI* the conformity index, *DI* the degree of inclusion, *DSC* Dice’s similarity coefficientm, ΔV: the distance between the COM of the targets

## Discussion

APBI, as a possible alternative to WBI, offers less overall treatment time and the delivery of a reduced dose to uninvolved portions of the breast and adjacent organs at risk [20, 21]. Undoubtedly, the irradiation of normal breast tissue would be decreased by reducing the EB-PBI target volume, which provides conditions for reducing toxicity or other side effects and improving the cosmetic outcome [22]. During the EB-PBI target definition, first and foremost is the identification and contouring of the GTV. MRI is recognized as an excellent imaging tool in diagnosing a primary breast tumor and has shown the ability to identify mammographically occult carcinoma [14, 15]. In the preoperative APBI study of van der Leij et al. [11], a virtual plan was made for preoperative EB-PBI, which resulted in a reduction in the GTV compared to that with postoperative EB-PBI. Hence, we aimed to clarify whether the delineation of the target volumes for postoperative prone EB-PBI might benefit from preoperative prone diagnostic MRI and to provide a reference for how to use preoperative diagnostic MRI to guide the delineation of target volumes for postoperative EB-PBI in the prone position.

Based on our analysis, the results showed that the GTV_postCT_ was significantly larger than the GTV_preMR_ by 12.58 cm^3^. Van der Leij et al. [11] also confirmed that a statistically significant difference was evident between the preoperative GTV and the postoperative tumor bed (7.71 cc lower in the preoperative EB-PBI target volume). In addition, our study demonstrated that the CTV_preMR + 10_ and PTV_preMR + 15_ were significantly smaller than the CTV_postCT + 10_ and PTV_postCT + 15_, respectively. Compared to the CTV_postCT + 10_ and PTV_postCT + 15_, the CTV_preMR + 20_ and PTV_preMR + 25_ were significantly greater by 51.81 cm^3^ and 43.99 cm^3^, respectively. Hence, if the expanded margin was too large, the preoperative EB-PBI would lose the advantage of reducing the dose to the ipsilateral breast, and the main factor to consider for the margin extension is the subclinical range. Controversy exists regarding EB-PBI treatment in terms of the subclinical range. Faverly et al. [23] have shown that a 10-mm tumor-free margin gives the best positive predictive value for breast cancers of limited extent. At present, a lumpectomy is performed with an intended macroscopic margin of at least 1.0 cm [24], and this value also represented a subclinical range that had been covered by previous studies [25, 26]. Therefore, it is resonable to reconstruct the CTV_MRI_ by adding a 1.0-cm margin around the GTV_MRI_. However, Schmitz et al. [13] indicated that typical treatment margins of 10 mm around the GTV_MRI_ might include occult disease in 52% of patients for MRI-guided BCT. When expanded with a 20-mm margin around the GTV_MRI_, a subclinical lesion could also be found in one-fourth of the patients. This might have been a consideration for van der Leij et al. [27] in delineating the CTV_MRI_ and PTV_MRI_ by expanding around the GTV_MRI_ with 20-mm and 25-mm margins, respectively. But, based on our result, the CTV_preMR + 20_ and PTV_preMR + 25_ were significantly greater, so the CTV_MRI_ and PTV_MRI_ by expanding around the GTV_MRI_ with 20-mm and 25-mm margins should not be advised. In our study, in comparison with the CTV_postCT + 10_ based on the postoperative TB, the CTV_MRI_ was not expanded by 15 mm around the GTV_preMR_; however, van der Leij et al.^11^ have shown that the difference was not statistically significant.

In theory, when there is equilateral excision around the primary tumor, there would be no significant difference between the CTV_preMR + 10_ and CTV_postCT + 10_, which are defined by expansion from the primary tumor based on preoperative MRI and by the postoperative TB, respectively. This seemingly contradictory difference can be explained by the asymmetric resection of the primary tumors [28]. If the anisotropic surgical margin caused by asymmetrical resection is taken into account, the volumes of the CTV_pre_ and CTV_post_ are comparable [11]. Furthermore, Zhang AP et al. [28] and den Hartogh et al. [29] indicated that because the majority of surgeons subjectively perform BCS based on palpation of the boundaries, which results in the asymmetric resection of primary tumors, neither the resection specimen volume nor the TB correlate with the visible tumor volume based on preoperative MRI. However, in our study, the Spearman rank correlation demonstrated that a statistically significant positive correlation exists between the GTV_preMR_ and GTV_postCT_ and between the CTV_preMR + 10_ and GTV_postCT._ This finding might be explained by the fact that in addition to the determination of the resection range based on palpation, the preoperative imaging data, such as MRI scans, have recently played an increasingly significant role in BCS, and the anisotropy between the specimen edge and tumor edge has been reduced.

MRI is not only the basis for the implementation of preoperative EB-PBI but also helpful for selecting patients suitable for postoperative EB-PBI, guiding postoperative target delineation for EB-PBI [30, 31]. The preoperative diagnostic MRI image was obtained in the prone treatment position, which is the same position as that of prone EB-PBI after BCS. Theoretically, DIR between the preoperative diagnostic MRI and postoperative prone CT simulation images should be conducive to the determination of the targets for postoperative prone EB-PBI. However, our study concluded that when based on the DIR of the thorax, the CI, DI and DSC were all poor for both the GTV_preMR_-GTV_postCT_ and CTV_preMR + 10_-GTV_postCT_ comparisons. The distances between the COMs of the GTV_preMR_-GTV_postCT_ and GTV_preMR + 10_-GTV_postCT_ were 2.71 cm and 2.67 cm, respectively. Moreover, the breast spatial matching between preoperative diagnostic MRI and postoperative CT simulation was not ideal, showing that the CI, DI and DSC values for the CTV_Breast-MRI_-CTV_Breast-CT_ did not reach 1, for perfect agreement between volumes.

The poor breast spatial matching might be mainly caused by the difference between the dedicated MRI bilateral breast coil and the dedicated treatment board for prone CT simulation. For preoperative diagnostic MRI, there are two apertures open on all sides to allow the bilateral breasts to hang freely away from the chest wall; however, the postoperative CT treatment board only contains an open aperture on one side, whereby only the ipsilateral breast can move away from the chest wall due to gravity in the prone position. Meanwhile, the contralateral breast is pulled away from the ipsilateral side by the baffle as much as possible, which might affect the natural overhang of the ipsilateral breast. Our results indicated that there was relatively poor spatial overlap between both the GTV_preMR_ and GTV_postCT_ and between the CTV_preMR + 10_ and GTV_postCT_. This result may be attributable to the poor breast spatial matching and could also be the result of the same spatial morphology among the GTV_preMR_, CTV_preMR + 10_ and GTV_postCT_ after DIR. Furthermore, from our analysis that was based on the DIR of the center-coincidence of the GTV_preMR_ and GTV_postCT_, the CI, DI and DSC values for the GTV_preMR_-GTV_postCT_ and the CTV_preMR + 10_-GTV_postCT_ were significantly improved compared with those based on the DIR of the thorax; however, these values were still poor. Therefore, it is unreasonable to use preoperative prone diagnostic MRI images to guide the postoperative target delineation for EB-PBI.

## Conclusions

Overall, in Chinese early-stage breast cancer patients enrolled to undergo prone EB-PBI, when defining the target based on the preoperative prone MRI images, the target volumes were significantly smaller when compared to those based on postoperative prone CT images. However, a statistically significant positive correlation was found between the MRI- and CT-based target volumes. Although based on DIR, there was relatively poor spatial overlap between the preoperative prone diagnostic MRI images and the postoperative prone CT simulation images for both the whole breast and the target volumes. Hence, it is unreasonable to use preoperative prone diagnostic MRI to guide postoperative target fusion delineation for EB-PBI. In fact, it is feasible to optimize the delineation of the postoperative EB-PBI target volumes by other means, such as clipping the surgical cavity by the surgical team in the presence of the radiation oncologist responsible for contouring for PBI and using respiratory gating with daily on board image verification before delivery of treatment can help in reducing the PTV margins. Further, studies on patterns of failure and adverse cosmetic outcome after EB-PBI can aid in refining the delineation techniques.

## Abbreviations

ALND: Axillary lymph node dissection
AP: Anterior-posterior
APBI: Accelerated partial breast irradiation
BCS: Breast-conserving surgery
BCT: Breast-conserving therapy
CI: Conformity index
COMs: Centers of mass
CT: Computed tomography
CTV: Clinical target volume
DI: Degree of inclusion
DIR: Deformable image registration
DSC: Dice’s similarity coefficient
EB-PBI: External-beam partial breast irradiation
GTV: Gross tumor volume
LR: Left-right
MRI: Magnetic resonance imaging
PTV: Planning target volume
si: Superior-inferior
SLND: Sentinel lymph node dissection
TB: Tumor bed
WBI: Whole breast irradiation
